# Identification and functional characterization of plasma exosomal miR-618 as a novel biomarker in recurrent depressive disorder

**DOI:** 10.3389/fphar.2026.1816232

**Published:** 2026-06-01

**Authors:** Xiangyang Zhang, Binbin Chen, Xue Yi, Meitao Duan, Chen Wang, Shuxian Li, Wei Wei

**Affiliations:** 1 College of Pharmacy, Jiamusi University, Jiamusi, China; 2 Fujian Psychiatric Center, Xiamen Xianyue Hospital Affiliated with Xiamen Medical College, Xiamen Xianyue Hospital, Fujian Clinical Research Center for Mental Disorders, Xiamen, Fujian, China; 3 Key Laboratory of Functional and Clinical Translational Medicine, Xiamen Medical College, Fujian Province University, Xiamen, China

**Keywords:** biomarkers, exosome, high-throughput sequencing, miRNA, recurrent depressive disorder, RT-qPCR

## Abstract

**Background:**

Given the substantial clinical and financial burden of Recurrent Depressive Disorder (RDD) and the critical lack of reliable biomarkers, this study investigated plasma exosomal microRNA (miRNA) as candidate biomarkers and therapeutic targets to overcome reliance on subjective diagnosis. The pathogenesis of RDD remains incompletely understood. To this end, we focused on miR-618 and conducted *in vivo* functional experiments to reveal its molecular mechanism in RDD.

**Methods:**

Differentially expressed miRNAs (DEmiRNAs) in plasma exosomes between RDD patients and healthy controls (HCs) were identified using high-throughput sequencing and reverse transcription-quantitative polymerase chain reaction (RT-qPCR). Receiver operating characteristic (ROC) curve analysis was used to evaluate their diagnostic value for RDD. Biological functions and pathways of DEmiRNA target genes were explored through Gene Ontology (GO) and Kyoto Encyclopedia of Genes and Genomes (KEGG) enrichment analyses. The miR-618 delivery vector was constructed by utilizing the exosomes secreted by HEK293 T cells. The molecular functions of miR-618 were verified through *in vivo* experiments.

**Results:**

High-throughput sequencing analysis identified 49 DEmiRNAs between the RDD patients and HCs. Subsequent validation via RT-qPCR demonstrated marked upregulation of miR-618 and miR-223–3p expression in plasma exosomes derived from RDD patients relative to HCs. ROC curve analysis indicated that miR-618 and miR-223–3p exhibited good diagnostic performance for RDD, with areas under the ROC curve (AUC) of 0.782 and 0.762, respectively. GO and KEGG pathway enrichment analyses indicated significant associations of DEmiRNAs target genes with key biological processes such as ER to Golgi ceramide transport and calcium ion export, and specific pathways including PI3K-Akt signaling pathway, MAPK signaling pathway and Wnt signaling pathway, etc. Functional validation *in vivo* indicated that miR-618 contributes to depression-like behaviors by regulateing the PI3K-Akt pathway and subsequently inducing neuronal and synaptic plasticity impairment.

**Conclusion:**

MiR-618 and miR-223–3p in plasma exosomes can serve as candidate non-invasive biomarkers for RDD. miR-618 contributes to depression-like behaviors by regulateing the PI3K-Akt pathway and subsequently inducing neuronal and synaptic plasticity impairment.

## 1 Introduction

Depressive disorder is a mood disorder primarily characterized by persistent depressive mood and anhedonia (Marx et al., 2023). As a mental disorder with high incidence rates and a long disease course, depressive disorder poses substantial risks, significantly impairing patients’ quality of life and posing serious threats to daily life, work, and life safety (Cui et al., 2024). The depressive disorder is frequently associated with recurrent episodes, demonstrating a relapse rate as high as 70% (Cosci et al., 2020). Recurrent Depressive Disorder (RDD) is a mood disorder characterised by two or more separate depressive episodes separated by symptom-free intervals lasting at least 2 months (Ding et al., 2022). During relapse phases, patients typically manifest symptoms including persistent low mood, diminished interest in life, chronic fatigue, psychomotor retardation, and fluctuating mental status (Cui et al., 2024). Compared to first-episode depression (FED), RDD patients exhibit significantly higher probabilities of suicidal behaviors, memory impairment, and terminal insomnia, which may constitute critical distinguishing features between RDD and FED (Huang et al., 2025). The current absence of reliable diagnostic biomarkers for RDD necessitates dependence on clinical interviews and behavioral observations—a subjective approach that increases the risk of delayed diagnosis or misdiagnosis. Therefore, the identification of biological markers for RDD represents an urgent clinical priority with significant implications.

Exosomes are a subtype of extracellular vesicles actively secreted by cells, with diameters ranging from 30 to 150 nm, and are ubiquitously present in various biological fluids, such as blood, urine, and saliva (Kalluri and LeBleu, 2020). Exosomes possess a stable double-layered membrane structure and can encapsulate a variety of bioactive molecules, such as nucleic acids (e.g., microRNAs) and proteins. This enables them to play a crucial role in intercellular communication and in the physiological and pathological processes of diseases like depressive disorder (Jiang et al., 2021; Yang et al., 2024). The protein and nucleic acid within exosomes reflect the functional status of their parent cells, offering a means to monitor cellular dynamics. Consequently, exosomes are increasingly recognized as highly promising biomarkers for disease diagnosis, prognostic evaluation, and therapeutic development (Jiang et al., 2021).

MicroRNAs (miRNAs) are a class of non-coding single-stranded RNA molecules with a length of approximately 18–25 nucleotides (Jing et al., 2020). As crucial regulatory elements in eukaryotic gene expression, miRNAs suppress mRNA translation or induce mRNA degradation through base-pair complementarity with target mRNAs, thereby regulating target genes (Naidu et al., 2015; Cuomo-Haymour et al., 2022; Diener et al., 2024). Exosomal miRNAs demonstrate significant potential in both diagnosis and treatment of depressive disorder. These miRNAs may serve as diagnostic biomarkers for depressive disorder due to their distinct expression patterns observed in patients with depressive disorders. Studies have identified significant upregulation of specific plasma miRNAs in Patients with depressive disorders, with these miRNAs exhibiting close associations with the pathophysiological processes of depressive disorder (Prodan-Bărbulescu et al., 2024a; Prodan-Bărbulescu et al., 2024b). Furthermore, exosomal miRNAs are considered as potential therapeutic agents because they can be transported across the blood-brain barrier into cerebral tissues. Targeted modulation of specific miRNAs could influence depressive disorder-related signaling pathways, thereby ameliorating clinical symptoms (Bhatt et al., 2021; van der Zee et al., 2022). However, current studies have not yet reported biomarkers for RDD, so the specific miRNAs associated with RDD remain to be elucidated.

In conclusion, exosomal miRNAs demonstrate promising potential as biomarkers and therapeutic targets in the diagnosis and treatment of depressive disorder. In this study, we investigated differentially expressed miRNAs (DEmiRNAs) in plasma exosomes of RDD patients versus matched healthy controls (HCs) to identify candidate miRNA biomarkers for RDD. Furthermore, through Gene Ontology (GO) and Kyoto Encyclopedia of Genes and Genomes (KEGG) pathway enrichment analyses, we explored the mechanism by which these DEmiRNAs regulate RDD pathophysiology. To investigate the role of miR-618 in RDD, we constructed an exosomal delivery vehicle using HEK293T cells to achieve targeted overexpression. Functional validation was performed *in vivo* to elucidate the associated molecular mechanisms of RDD. These findings will be of great value for advancing diagnostic strategies and personalised treatment for RDD.

## 2 Materials and methods

### 2.1 Participants and samples

RDD patients who were hospitalised in Xiamen Xianyue Hospital were selected as study subjects. Inclusion criteria: (1) recurrent depressive disorder met the relevant diagnostic criteria in Chinese Classification of Mental Disorders, Third Edition (CCMD-3) or International Classification of Diseases, 10th Revision (ICD10); (2) 17-item Hamilton Depression Scale (HAMD-17) score ≥17; (3) aged 18–65 years old; and (4) signed an informed consent form. Exclusion criteria: (1) undergoing hormone therapy; (2) history of psychoactive substance use and alcoholism; (3) combination of severe physical disease or organic brain disease; (4) during pregnancy or lactation; and (5) other psychiatric diseases such as bi-directional affective disorder, schizophrenia, epilepsy, etc. HCs matched with patients’ age and gender were recruited from the health checkups of Xiamen Xianyue Hospital during the same period. Inclusion criteria: not having been diagnosed with any psychiatric disease, and no family history of psychiatric disease, major somatic disease, or neurodevelopmental disorder.

A total of 42 participants were recruited for this study, divided into 21 patients with RDD (RDD group) and 21 HCs (HC group). In each group, three plasma samples were used for high-throughput sequencing, while the remaining 18 samples were used for reverse transcription-quantitative polymerase chain reaction (RT-qPCR). Demographic information about each study subject was collected using a general information questionnaire, and two experienced psychiatrists assessed the level of depression and anxiety of each study subject using the HAMD-17 and the Hamilton Anxiety Scale (HAMA) (Turpeinen and Hämäläinen, 2013). There was no statistically significant difference between the RDD group and the HC group in terms of age and gender (Table 1). Venous blood from participants who had fasted for 8 h was collected in EDTA blood collection tubes between 8 and 10 a.m. After standing for 10 min at room temperature, the tubes were centrifuged at 3,000 rpm for 10 min in a 4 °C centrifuge to collect plasma, which was then stored in a −80 °C refrigerator.

**TABLE 1:** Demographic and clinical characteristics for RDD and HCs.

​	RDD (n = 21)	HCs (n = 21)	P-value
Females (%)	66.7	66.7	1
Age (year)	24.19 ± 4.21	23.81 ± 3.06	0.739
HAMD scores	21.83 ± 2.33	8.28 ± 1.87	<0.0001
HAMA scores	12.11 ± 2.61	7.33 ± 1.50	<0.0001

This study involving human participants was reviewed and approved by the Medical Ethics Committee of the Second Affiliated Hospital of Xiamen Medical College (Approval number: 2,025,088, Apr. 3, 2025), and all participants signed an informed consent form before participation. The study was conducted in accordance with the Declaration of Helsinki and all applicable laws and institutional guidelines.

### 2.2 Exosome isolation and characterization

Exosomes were extracted and purified from 500 µL of plasma using the Exosome Isolation and Purification Kit Plus (Umibio, China), following the manufacturer’s instructions. Extracted exosomes were characterised using several complementary methods, namely, transmission electron microscopy (TEM), nanoparticle tracking analysis (NTA), and Western blot, according to the Guidelines of the International Society for Extracellular Vesicles (ISEV) (Lötvall et al., 2014).

#### 2.2.1 TEM

10 μL of exosome suspension was added on a carbon-coated Cu grid (ProSciTech, Australia), followed by blotting out the excess liquid on the Cu grid with filter paper after 2 min. The Cu grid were then negatively stained with 10 μL of 2% Phosphotungstic acid (YEASEN, China), 2 min later, the excess liquid was blotted out with filter paper before the Cu grids were transferred to infrared light for drying. The Cu grid with fixed sample were placed on a Hitachi HT7700 EXALEN (Hitachi, Japan) with an accelerating voltage of 120 kV to observe the morphology of exosomes and take photographs.

#### 2.2.2 NTA

The particle size and particle concentration of exosomes were detected using a Zeta View Nanoparticle Tracking Analyser (Particle Metrix, Germany). Firstly, 10 μL of 100 nm polystyrene standard solution was diluted 250,000 fold using ultrapure water to obtain the calibration solution, which was injected into the instrument using a syringe in order to achieve the calibration of the instrument. Then 20 μL of exosome suspension was added to 6 mL of phosphate buffer saline (PBS, Servicebio, China) before 5 mL of 300-fold diluted exosome suspension was injected into the instrument using a syringe for detection.

#### 2.2.3 Western blot

100 μL of exosome suspension was added with 100 μL of RIPA lysis buffer containing 1 mM PMSF (LABLEAD, China). After 30 min of lysis on ice with continuous shaking, the mixture was centrifuged at 12,000 × g for 10 min (4 °C). The supernatant was then carefully transferred to a fresh 1.5 mL centrifuge tube. The supernatant contained total protein who’s concentration was determined using the BCA Protein Concentration Assay Kit (LABLEAD, China). The remaining protein samples were mixed with 5× SDS-PAGE protein loading buffer (Servicebio, China) at a 4:1 (v/v) ratio and heat-denatured at 95 °C for 5 min using a metal heating block.

40 μg of protein was separated on a 10% SDS-PAGE gel (pre-cast using a One Step PAGE Gel Super Fast Preparation Kit, Meilun, China) by sodium dodecyl sulfate polyacrylamide gel electrophoresis (SDS-PAGE) under constant voltage (200 V) for 45 min, followed by being electrophoretically transferred to a polyvinylidene fluoride (PVDF) membrane (Immobilon, Germany) at 400 mA for 25 min. After incubation for 1 h at room temperature with Tris-Buffered Saline with Tween 20 (TBST, Servicebio, China) containing 5% Bovine Serum Albumin (Beyotime, China), the PVDF membrane was rinsed three times with TBST for 10 min each, followed by overnight incubation at 4 °C with the following primary antibody: CD9 Recombinant Rabbit Monoclonal Antibody (clone NO. SA35-08, Cat# ET1601-9, 1:1,000, HUABIO, China), CD63 Recombinant Rabbit Monoclonal Antibody (clone NO. SY21-02, Cat# ET1607-2, 1:500, HUABIO, China) and TSG101 Recombinant Rabbit Monoclonal Antibody (clone NO. JJ0900, Cat# ET1701-59, 1:2000, HUABIO, China). The following day, the PVDF membrane was rinsed three times with TBST for 10 min each, and then incubated with HRP-Labelled Goat Anti-Rabbit IgG (H + L) (Cat# A0208, 1:1,000, Beyotime, China) for 1 h at room temperature. After incubation, the PVDF membrane was rinsed again three times with TBST for 10 min each. Finally, the PVDF membrane was infiltrated with BeyoECL Plus (Beyotime, China), followed by a protein blotting assay using the e-BLOT Touch Imager System (e-BLOT, China).

### 2.3 MiRNA sequencing and differentl expression analysis

Total RNA was extracted from exosomes using the Exosome RNA Purification Kit (EZBioscience, United States). After that, miRNA high-throughput sequencing was performed by Cloud-Seq Biotech (Shanghai, China). Cloud-Seq Biotech analyzed the raw sequencing data using established methods documented in prior literature (Huang et al., 2022). See the Supplementary Material for details.

### 2.4 PPI network construction and screening of hub genes

Target gene prediction for DEmiRNAs was performed through the miRWalk, TargetScan, and miRDB database, with the final target set defined by the intersection of all three predictions. Disease-associated targets for RDD were concurrently identified using the GeneCards database. The potential miRNA-regulated targets in RDD pathogenesis were determined by intersecting the DEmiRNA targets with RDD-related genes. These candidate genes were then analyzed for protein-protein interactions (PPI) using the STRING database, applying a confidence score threshold of >0.4 for interaction inclusion. Isolated protein nodes not connected to the primary interaction network were excluded to enhance biological relevance. The PPI network was visualized using Cytoscape 3.10.0, followed by hub gene identification through the CytoHubba plugin.

### 2.5 GO and KEGG pathway enrichment analysis

To better explain the relationship between DEmiRNA and RDD, we performed GO and KEGG pathway enrichment analysis for DEmiRNA target genes to speculate on the potential mechanisms by which DEmiRNAs regulate RDD. All target genes were entered into the DAVID database for GO and KEGG pathway enrichment analysis, with P-value ≤0.05 as the threshold for significant enrichment.

### 2.6 RT-qPCR

100 ng of total RNA from each exosome sample was used for RT-qPCR. This study employed the stem-loop dye method for RT-qPCR analysis of miRNAs. MiRNAs were reverse transcribed into template DNA using the BIOG miRNA Stem-loop RT Kit (BAIDAI, China), with small nuclear RNA U6 serving as the endogenous control. Subsequently, the qPCR reaction mixture was prepared in a 96-well PCR plate (Service, China) using the BIOG miRNA Stem-loop SYBR qPCR Kit (BAIDAI, China). Three technical replicates were set for each gene. The reaction program outlined in Table 2 was programmed into the LightCycler 96 Real-Time PCR System (Roche, Switzerland) to perform qPCR and acquire fluorescence data. Relative expression levels of miRNAs were determined using the 2^−ΔΔCq^ method (Xie et al., 2020). See the Supplementary Material for details. The primers employed in this experiment were purchased from Baidai Biotechnology Co., Ltd., and their sequences are summarized in Table 3.

**TABLE 2:** qPCR reaction procedure.

Preincubation	95 °C for 2 min
2 step amplification	95 °C for 10 s; 60 °C for 30 s; 40 cycles
Melting	95 °C for 10 s; 65 °C for 60 s; 97 °C for 1 s
Cooling	37 °C for 30 s

**TABLE 3:** Primer information.

Gene	RT primer	Forward primer	Reverse primer
U6	AAC​GCT​TCA​CGA​ATT​TGC​GT	CTTGCTTCGGCAGCACA	AAC​GCT​TCA​CGA​ATT​TGC​GT
Hsa-miR-223–3p	GTC​GTA​TCC​AGT​GCA​GGG​TCC​GAG​GTA​TTC​GCA​CTG​GAT​ACG​ACT​GGG​GT	GCG​CGT​GTC​AGT​TTG​TCA​AAT	AGT​GCA​GGG​TCC​GAG​GTA​TT
Hsa-miR-618	GTCGTATCCAGTGCAGGGTCCGAGGTATICGCACTGGATACGACACTCAG	GCG​CGA​AAC​TCT​ACT​TGT​CCT​T	AGT​GCA​GGG​TCC​GAG​GTA​TT
Hsa-let-7b-5p	GTC​GTA​TCC​AGT​GCA​GGG​TCC​GAG​GTA​TTC​GCA​CTG​GAT​ACG​ACA​ACC​AC	GCT​CGT​GAG​GTA​GTA​GGT​TGT	AGT​GCA​GGG​TCC​GAG​GTA​TT
Hsa-miR-203a-3p	GTC​GTA​TCC​AGT​GCA​GGG​TCC​GAG​GTA​TTC​GCA​CTG​GAT​ACG​ACC​TAG​TG	CGC​CGT​GAA​ATG​TTT​AGG​AC	AGT​GCA​GGG​TCC​GAG​GTA​TT
Hsa-miR-451a	GTC​GTA​TCC​AGT​GCA​GGG​TCC​GAG​GTA​TTC​GCA​CTG​GAT​ACG​ACA​ACT​CA	GGC​GAA​ACC​GTT​ACC​ATT​ACT	AGT​GCA​GGG​TCC​GAG​GTA​TT

### 2.7 Cell culture

The HEK293 T and BV2 cell lines were purchased from Shanghai Jinyuan Biotechnology Co., Ltd. Both cell types were cultured in T75 flasks (LABLEAD, China) with 10 mL of DMEM medium (MeilunBio, China), supplemented with 10% fetal bovine serum (FBS) (Gibco, United States) and 100 U/mL penicillin-streptomycin (MeilunBio, China), and incubated at 37 °C in an incubator with 5% CO_2_ atmosphere.

### 2.8 Cell transfection

According to the manufacturer’s instructions, HEK293 T cells cultured in T75 flasks were transfected with cy3-labeled miR-NC mimics or cy5-labeled miR-618 mimics (synthesized by Fuzhou Zaiji Biotechnology Co., Ltd.) using Lipo293 Plus Transfection Reagent (Byotime, China). Control cells did not undergo transfection. 6 h later, the medium was replaced with 10 mL of fresh DMEM containing 10% exosome-depleted FBS (Umibio, China). After a further 24-h incubation, the cell culture supernatant was collected for exosome extraction. See the Supplementary Material for details.

### 2.9 Verification of cell transfection efficiency

An appropriate number of HEK293T cells were seeded into confocal dishes (NEST, China) and cultured overnight. When the cells reached approximately 70% confluence the next day, they were transfected with Cy3-labeled miR-NC mimics or Cy5-labeled miR-618 mimics using Lipo293 Plus Transfection Reagent, according to the manufacturer’s instructions. Control cells did not undergo transfection. After an additional 6 h of incubation, cells were fixed with 4% paraformaldehyde (Biosharp, China), counterstained with DAPI solution (10 μg/mL, Meilunbio, China), and then imaged under a laser scanning confocal microscope (Leica, Germany). See the Supplementary Material for details.

### 2.10 Exosome extraction and characterization

After collecting 20 mL of cell culture supernatant from each of the three groups—untreated HEK293 T cells, cells transfected with cy3-labeled miR-NC mimics, and cells transfected with cy5-labeled miR-618 mimics, exosomes were isolated and purified using a Cell Culture Supernatant Exosome Extraction Kit (Umibio, China) according to the manufacturer’s instructions. The resulting exosome samples were designated as Exo, miR-NC Exo, and miR-618 Exo, respectively. The harvested exosomes were characterized by TEM, NTA, and Western blot. See the Supplementary Material for details.

### 2.11 Cell uptake

An appropriate number of BV2 cells were seeded into confocal dishes and cultured overnight. When the cells reached approximately 70% confluence the following day, they were treated with 200 μg/mL of Exo, miR-NC Exo, or miR-618 Exo, respectively. After 24 h of incubation, the cells were fixed and stained following the procedure described in the previous section. Finally, the dishes were imaged under a laser scanning confocal microscope.

### 2.12 Animals and drug administration

Male 6-week-old C57BL/6J mice (specific pathogen-free) were purchased from Xiamen Fudexin Biotechnology Co., Ltd (license No. SCXK 2025-0001). All mice were housed in the animal facility of the Xiamen Medical College Basic Research Platform under controlled conditions: temperature 23 °C ± 2 °C, humidity 50% ± 1%, a 12 h light/dark cycle, and free access to food and water. All animal experiments were approved by the Medical Ethics Committee of Xiamen Medical College (Approval number: 20251030034, Oct. 30, 2025)and conducted in accordance with the relevant guidelines and regulations for animal experimentation. All animal experiments comply with the “3 R” principle.

After 1 week of acclimation, the mice were randomly divided into three groups: control, miR-NC, and miR-618 groups. Mice received tail vein injections of exosomes every 3 days for a total of four administrations at a dose of 10 mg/kg. The Control group was injected with Exo, the miR-NC group with miR-NC Exo, and the miR-618 group with miR-618 Exo. Behavioral tests were initiated on the day following the final injection.

### 2.13 Sucrose preference test

The Sucrose Preference Test (SPT) was performed to assess anhedonia-like behavior in mice, following previously described methods with minor modifications (Shang et al., 2017). Briefly, mice were first subjected to a 72-h training period under single-housing conditions. During the first 24 h, only 1% sucrose water was provided. For the subsequent 48 h, both 1% sucrose water and regular drinking water were made available, with the positions of the two bottles interchanged every 12 h to prevent place preference. After training, mice were deprived of food and water for 24 h. The test was then conducted by simultaneously presenting each singly housed mouse with pre-weighed bottles containing 1% sucrose water and regular drinking water. Consumption from each bottle was recorded over a 24-h period, then the proportion of sucrose solution consumed was calculated.

### 2.14 Open field test

Locomotor activity and anxiety-like behavior were assessed using the open field test (OFT) following the method described in the literature (Shang et al., 2017). Briefly, each mouse was gently placed in a fixed corner of a white opaque open-field arena (40 × 40 × 40 cm). Its movement was recorded for 10 min and analyzed using the Tracking Master behavioral analysis system (Beijing Zhongshi Technology Co., Ltd.).

### 2.15 Forced swim test

Despair-like behavior was further examined using the forced swim test (FST) according to published methods (Shang et al., 2017). The FST was performed by placing each mouse individually into a transparent cylindrical tank filled with water (24 °C) to a depth of 15 cm. The test lasted 6 min, and immobility time during the final 4 min was recorded and analyzed using the Tracking Master behavioral analysis system.

### 2.16 RT-qPCR

Following anesthesia, mice were euthanized by decapitation on ice. The brains were rapidly dissected and placed in pre-chilled PBS. Total RNA, including miRNA, was isolated from the hippocampus using the MiPure Cell/Tissue miRNA Kit (Vazyme, China) according to the manufacturer’s protocol.

For miRNA quantification, RT-qPCR was performed as previously described. For mRNA analysis, RT-qPCR was conducted using the BeyoFast SYBR Green One-Step qRT-PCR Kit (Byotime, China). Briefly, reaction mixtures were prepared in a 96-well PCR plate following the composition outlined in Table 4, with β-actin used as the internal reference. The plate was then loaded into a LightCycler 96 real-time PCR system for amplification and fluorescence signal detection with the reaction program outlined in Table 5. The relative expression levels of the target genes were calculated using the 2^−ΔΔCq^ method. The primers used for mRNA quantification were purchased from Beijing Dingguochangsheng Biotechnology Co., Ltd., and their sequences are listed in Table 6.

**TABLE 4:** qPCR reaction procedure.

Reagent	Volume
SYBR green one-step reaction buffer (2X)	10 µL
SYBR green one-step enzyme mix (10X)	2 µL
Forward and reverse primer mix (2.5 μM each)	2 µL
Template RNA	300 ng
RNase-free water	Up to 20 μL

**TABLE 5:** qPCR reaction procedure.

Preincubation	50 °C for 30 min; 95 °C for 5 min
3 step amplification	95 °C for 15 s; 60 °C for 30 s; 72 °C for 30 s; 40 cycles
Melting	95 °C for 10 s; 65 °C for 60 s; 97 °C for 1 s
Cooling	37 °C for 30 s

**TABLE 6:** Primer sequences.

Gene	Forward primer	Reverse primer
TP53	TGT​ATG​CCG​AGT​ATG​TGG​AAG​A	CAG​TGT​GAT​GGT​AAG​GAT​AGG
CCND1	GAG​AAG​TTG​TGC​ATC​TAC​ACT​G	AAA​TGA​ACT​TCA​CAT​CTG​TGG​C
β-actin	CGTCTTCCCCTCCATCG	CTCGTTAATGTCACGCAC

### 2.17 Western blot

The hippocampus was homogenized in pre-chilled RIPA lysis buffer containing 1 mM PMSF. The homogenates were then centrifuged at 12,000 × g for 20 min at 4 °C. The resulting supernatant, containing the total protein fraction, was collected for subsequent analysis. The protein concentration of each sample was determined using the BCA Protein Concentration Assay Kit, following the manufacturer’s instructions. Subsequently, protein samples were mixed with one-fourth volume of 5× SDS-PAGE protein loading buffer and denatured by heating at 95 °C for 5 min.

For immunoblotting, 20 μg of protein were separated by electrophoresis on a 10% SDS-PAGE gel. The separated proteins were then electrophoretically transferred onto a PVDF membrane. Following transfer, the membrane was blocked with 5% bovine serum albumin in TBST for 1.5 h at room temperature. The blocked membrane was incubated with the appropriate primary antibodies overnight at 4 °C. After extensive washing with TBST, the membrane was incubated with HRP-Labelled Goat Anti-Rabbit IgG (H + L) for 1 h at room temperature. Protein bands were visualized using an e-BLOT Touch Imager System. See the Supplementary Material for details.

### 2.18 Hematoxylin-eosin staining

Following anesthesia, mice were transcardially perfused with ice-cold physiological saline before brain extraction. Harvested brains were immediately fixed in 4% paraformaldehyde at room temperature for 24 h, then sequentially dehydrated through an ethanol gradient. Tissues were paraffin-embedded and sectioned coronally at 5 μm thickness using a rotary microtome. For histological analysis, the sections were stained with 0.5% hematoxylin (Servicebio, China) for 5 min and 0.05% eosin (Servicebio, China) for 3 min at room temperature, then examined under bright-field microscopy for pathological assessment.

### 2.19 Statistical analysis

All statistical analyses were performed using the software Graphpad Prism 10.1.2 (GraphPad Software, United States). In demographic information, data were presented as mean ± standard deviation (SD). Student’s t-test was applied for the comparison of two groups. The comparisons among multiple groups were conducted using one-way analysis of variance. Receiver operating characteristic (ROC) curve analysis was conducted using SPSS Statistics 27 (IBM, United States). The Pearson correlation analysis was used to assess the correlation between the two variables. P < 0.05 was considered statistically significant.

## 3 Results

### 3.1 Plasma exosome characterization

**FIGURE 1:**
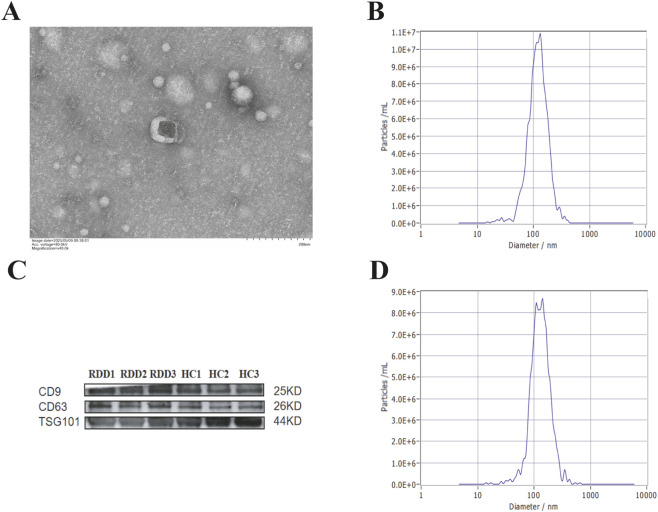
Characterisation of plasma exosomes. **(A)** The morphology of exosomes observed by TEM. Scale bar = 200 nm. **(B)** The particle sizes of plasma exosomes in the HC group. **(C)** Exosome marker proteins detected by Western blot. **(D)** The particle sizes of plasma exosomes in RDD group.

The extracted exosomes need to be characterised according to the recommendation of ISEV to confirm that the extracted particles are exosomes. Therefore, we characterised the extracted exosomes from three perspectives: morphology, particle size analysis, and marker proteins. First, plasma exosomes were observed using TEM. The exosomes were about 100 nm in size and showed a cup shape (Figure 1A), which was consistent with the morphological characteristics of exosomes. In addition, NTA was performed to analyse the particle size and particle concentration of the exosome suspensions. The particle sizes of plasma exosomes in the HC and RDD groups ranged from 50 to 300 nm (Figures 1B,D), with the peak particle sizes of 131 nm and 141 nm, and the particle concentrations of 8.2 × 10^10^ Particles/mL and 6.6 × 10^10^ Particles/mL. In this study, plasma exosome marker proteins CD9 (25KD), CD63 (26KD), and TSG101 (44KD) were detected in the RDD and HC groups by Western blot, and the bands of the target proteins were clearly visible (Figure 1C). The above three experiments demonstrated that exosomes were successfully extracted.

### 3.2 Differential expression analysis

This study detected the expression of exosomal miRNAs in 3 cases of RDD and 3 cases of HCs through high-throughput sequencing. For sequencing raw data, fold change, P-value, and FDR were calculated using the normalised number of reads, with fold change ≥2.0 and P-value ≤0.05 as the DEmiRNA screening threshold. 49 DEmiRNAs (P < 0.05, fold change ≥2, including 10 known and 39 newly discovered miRNAs) were found between the RDD and HC groups, of which 5 miRNAs were upregulated, and 44 miRNAs were downregulated (Supplementary Table S1). 10 known DEmiRNAs were screened for subsequent studies.

**FIGURE 2:**
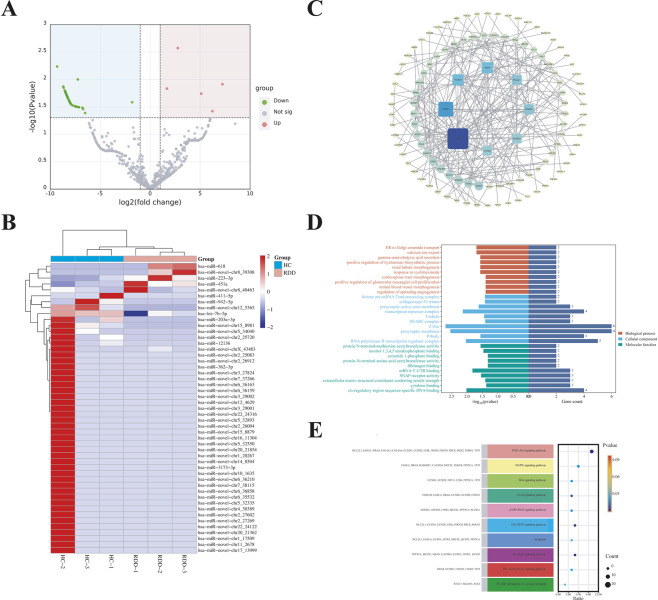
Differential expression and bioinformatics analysis of miRNAs. **(A)** Volcano plot of DEmiRNAs between RDD and HC groups. Each point represents a detected miRNA, the horizontal axis is log_2_ (fold change), where the more off-centre the point is, the greater the fold difference is; the vertical axis is -log_10_(P-value), where a greater value on the vertical axis indicates a more significant difference. Therefore, the green point in the upper left corner and the red point in the upper right corner of the volcano plot indicate the downregulated miRNAs and upregulated miRNAs with significant differences in the RDD group, respectively. **(B)** Clustering heatmap of DEmiRNAs between RDD and HC groups. The horizontal coordinates in the graph represent the names of the samples, and the vertical coordinates indicate the DEmiRNAs and their clustering in the samples. Red color indicates high expression and blue color indicates low expression. **(C)** PPI network of target genes of DEmiRNAs. The larger the protein node, the darker the color, representing the more critical it is. The eight genes in the innermost circle are hub genes. **(D)** Bar plot of GO enrichment analysis between the RDD and HC groups. **(E)** Sankey bubble plot of the top 10 related KEGG pathways between the RDD and HC groups.

In order to more visually demonstrate the up- and downregulation of miRNAs in the RDD group, a volcano plot (Figure 2A) was generated through the CNSknowall (https://cnsknowall.com). To visualise the expression of DEmiRNAs in each sample, a clustering heatmap (Figure 2B) was plotted through the SRplot (https://www.bioinformatics.com.cn) (Tang et al., 2023). The heatmap revealed DEmiRNAs expression patterns between the RDD and HC groups.

### 3.3 PPI network and hub genes

We predicted 193 mRNAs interacting with DEmiRNA that were simultaneously recorded in the miRWalk, TargetScan, and miRDB databases. A total of 10,055 RDD-associated targets were predicted in the Genecards database. After taking the intersection of these two sets, the final list comprised 137 target genes. STRING database was used to analyse the interaction relationship between target gene proteins, followed by a PPI network (Figure 2C) construction generated by Cytoscape 3.10.0 software. The Cytohubba plugin was employed to identify key nodes within the protein interaction network, then the top eight genes ranked by degree value were selected as hub genes, including TP53, CCND1, CCND2, FOXO1, NRAS, BCL2L1, SCARB1, and MAD2.

### 3.4 GO and KEGG pathway enrichment analysis

GO and KEGG pathway analysis of target genes were performed by the DAVID database before 69 biological processes, 30 cellular components, 31 molecular functions, and 33 signalling pathways were obtained. The top 10 most significantly enriched (Fold Enrichment top 10) functional entries in each GO classification were displayed on the bar plot (Figure 2D), which was generated by SRplot. Target genes are mainly involved in biological processes, including ER to Golgi ceramide transport and calcium ion export, *etc.*,; cellular components, including histone pre-mRNA 3′end processing complex and collagen type IV trimer, *etc.*,; molecular functions, including protein N-terminal-methionine acetyltransferase activity and inositol 1,3,4,5 tetrakisphosphate binding, etc.

The top 10 pathways most highly associated with MDD were selected to be displayed on the Sankey bubble plot (Figure 2E), which was generated through CNSknowall. DEmiRNAs were mainly enriched in pathways such as PI3K-Akt signaling pathway, MAPK signaling pathway, and Wnt signaling pathway, etc.

### 3.5 RT-qPCR

**FIGURE 3:**
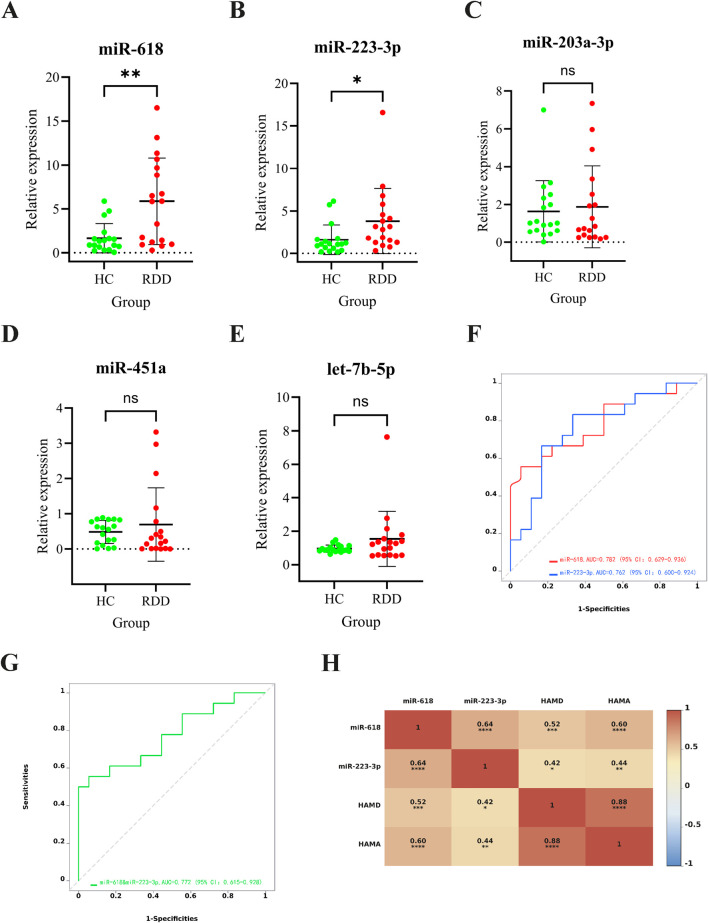
Association between miR-618 and miR-223–3p and RDD. The relative expression levels of **(A)** miR-618, **(B)** miR-223-3p, **(C)** miR-203a-3p, **(D)** miR-451a, and **(E)** let-7b-5p (relative to U6) in plasma exosomes were determined by RT-qPCR in the RDD group (n = 18) and HC group (n = 18). **(F)** ROC curves for miR-618 and miR-223-3p. **(G)** A combined diagnostic model of miR-618 and miR-223-3p constructed based on ROC curve analysis. **(H)** The Pearson correlation analysis between the dysregulated miRNAs and clinical Hamilton Rating Scales scores. *P < 0.05, **P < 0.01, ***P < 0.001, ****P < 0.0001, ns indicates not significant.

To validate the results of miRNA-seq, plasma exosome samples from 18 RDD patients and 18 HCs were analyzed using RT-qPCR to evaluate the expression levels of five selected miRNAs (miR-618, miR-223–3p, miR-451a, miR-203a-3p, and let-7b-5p). Among the selected miRNAs, the average expression levels of miR-618 and miR-223–3p in the RDD group were significantly higher than those in the HC group (Figures 3A,B), which was consistent with the high-throughput sequencing results. However, no statistically significant differences were observed in the expression levels of miR-451a, miR-203a-3p, and let-7b-5p between the two groups (Figures 3C–E).

### 3.6 Diagnostic capability and correlation analysis of dysregulated miRNAs

Based on the results of RT-qPCR, the potential diagnostic value of the significantly differentially expressed miRNAs was further evaluated using ROC curve analysis. The area under the ROC curve (AUC) for miR-618 was 0.782 (95% CI = 0.629–0.936, p < 0.05, sensitivity = 50.0%, specificity = 93.7%, Figure 3F), while that for miR-223–3p was 0.762 (95% CI = 0.600–0.924, p < 0.05, sensitivity = 85.0%, specificity = 62.5%, Figure 3F). Both miR-618 and miR-223–3p demonstrated good diagnostic performance, indicating their potential as biomarkers for RDD diagnosis. Furthermore, a combined diagnostic model of miR-618 and miR-223–3p was established, with an AUC of 0.772 (95% CI = 0.615–0.928, p < 0.05, sensitivity = 45.0%, specificity = 100.0%, Figure 3G). The combined diagnostic performance of miR-618 and miR-223–3p was slightly lower than that of miR-618 alone. Furthermore, the correlation between the expression levels of dysregulated miRNAs and clinical Hamilton Rating Scales scores was analyzed using the Pearson correlation coefficient (*r*). The results revealed a positive correlation between the expression levels of miR-618 and miR-223–3p (*r* = 0.64). Additionally, both miRNAs exhibited positive correlations with HAMD or HAMA scores (Figure 3H).

### 3.7 Cell transfection

**FIGURE 4:**
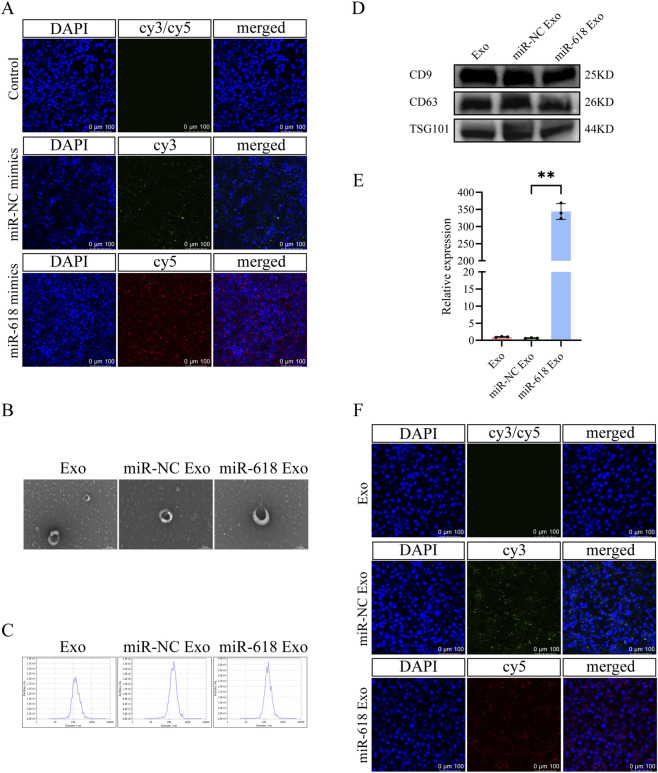
Construction of miR-618 delivery vector. **(A)** Successful transfection of miR-618 into HEK293T cells was confirmed. Scale bar = 100 μm. **(B)** The morphology of exosomes observed by TEM. Scale bar = 200 nm. **(C)** The particle sizes of exosomes. **(D)** Exosome marker proteins detected by Western blot. **(E)** The levels of miR-618 in exosomes were determined by RT-qPCR. **P < 0.01. **(F)** Uptake of exosomes loaded with miR-618 mimics by BV2 cells was observed. Scale bar = 100 μm.

Transfection efficiency was evaluated by fluorescence co-localization. Strong green or red signals surrounding the DAPI-stained nuclei confirmed successful delivery of miR-NC mimics or miR-618 mimics in most HEK293 T cells (Figure 4A).

### 3.8 Characterization of exosomes derived from cell supernatant

Exosomes isolated from untreated or transfected cells were characterized. TEM revealed the classic cup-shaped morphology of exosomes, with a diameter of approximately 100 nm (Figure 4B). NTA indicated that the particle size distribution ranged from 50 to 300 nm, consistent with typical exosomal dimensions (Figure 4C). Exosome marker proteins CD9 (25KD), CD63 (26KD), and TSG101 (44KD) were detected by Western blot, and the bands of the target proteins were clearly visible (Figure 4D). The above three experiments demonstrated that Cell supernatant exosomes were successfully extracted.

### 3.9 RT-qPCR

We measured the level of miR-618 in Exo, miR-NC Exo, and miR-618 Exo by RT-qPCR. Compared with Exo and miR-NC Exo, the loading amount of miR-618 mimics in miR-618 Exo was significantly increased (Figure 4E).

### 3.10 Cell uptake

Uptake of exosomes by BV2 cells was assessed via fluorescence co-localization analysis. The presence of extensive green or red fluorescent signals surrounding the DAPI-stained nuclei (blue) (Figure 4F) indicated that the exosomes were successfully loaded with either miR-NC mimics or miR-618 mimics and subsequently internalized by the BV2 cells.

### 3.11 Behavioral test

**FIGURE 5:**
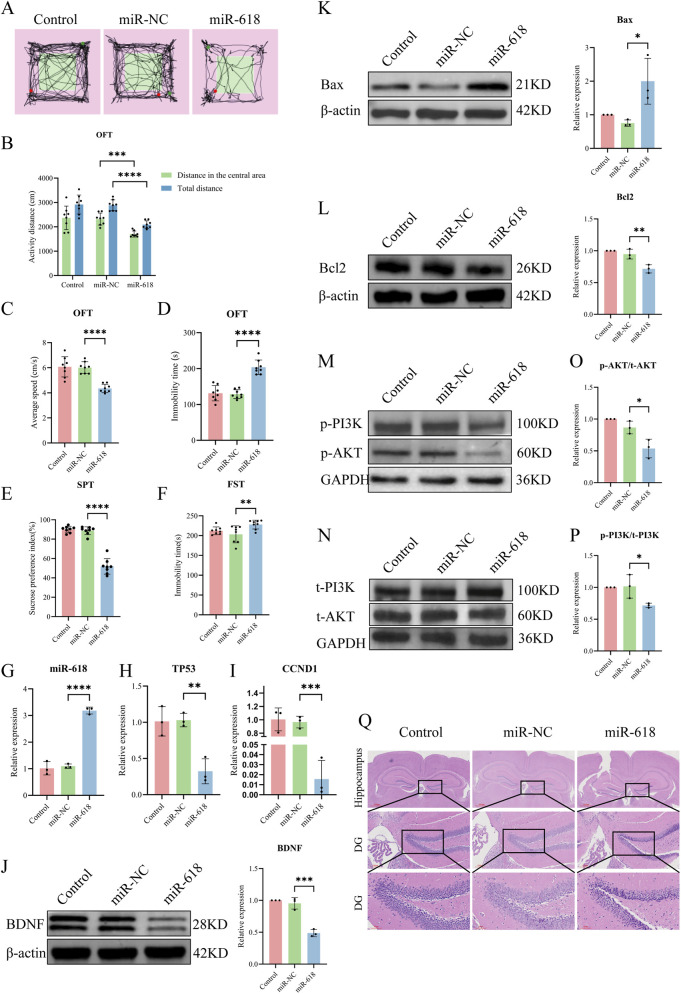
Functional validation of miR-618. **(A)** The representative activity trajectory of the mice in the OFT. **(B)** The activity distance of the mice in the OFT. **(C)** The average speed of the mice in the OFT. **(D)** The immobility time of the mice in the OFT. **(E)** The sucrose preference index of the mice in the SPT. **(F)** The immobility time of the mice in the FST. **(G–I)** The relative expression levels of miR-618 **(G)**, TP53 **(H)**, and CCND1 **(I)** in the hippocampal tissues were examined by RT-qPCR. **(J–N)** The representative immunoblotting band in the Western blot. The expression levels of BDNF **(J)**, Bax **(K)**, and Bcl2 **(L)** were normalized to β-actin. The expression levels of phosphorylated Akt **(O)** and phosphorylated PI3K **(P)** were calculated relative to their respective total protein levels. *P < 0.05, **P < 0.01, ***P < 0.001, ****P < 0.0001. **(Q)** Representative images of the DG stained with hematoxylin and eosin. Scale bars (from top to bottom): 600 μm, 100 μm, 50 µm.

In the OFT, compared with the miR-NC group, the miR-618 group showed a significant decrease in activity distance (Figures 5A,B) and average speed (Figure 5C), along with a significant increase in immobility time (Figure 5D); however, the control and miR-NC groups did not differ significantly on these measures. In the SPT, the sucrose preference index was significantly lower in the miR-618 group than in the miR-NC group, while no significant difference was detected between the control and miR-NC groups (Figure 5E). Similarly, in the FST, immobility time was significantly longer in the miR-618 group relative to the miR-NC group, whereas again no significant difference was observed between the control and miR-NC groups (Figure 5F).

### 3.12 RT-qPCR

The relative expression levels of miR-618, TP53, and CCND1 in the hippocampal tissues were examined by RT-qPCR. Compared with the miR-NC group, the miR-618 group exhibited a significant upregulation of miR-618 (Figure 5G) and significant down-regulations of TP53 (Figure 5H) and CCND1 (Figure 5I). No significant differences in the expression of these genes were observed between the control and miR-NC groups.

### 3.13 Western blot

The protein expression levels of key molecules in the PI3K-Akt signaling pathway, apoptosis‐related proteins (Bax and Bcl2), and the synaptic plasticity marker BDNF in the hippocampal tissues were analyzed by Western blot. Compared with the miR‐NC group, the miR‐618 group showed significantly decreased ratios of p‐PI3K/t‐PI3K and p‐Akt/t‐Akt (Figures 5M-P), as well as significantly reduced expression of BDNF (Figure 5J) and Bcl2 (Figure 5L), while the expression of Bax was markedly increased (Figure 5K). In contrast, no significant differences in the expression of these proteins were observed between the control and miR‐NC groups (Figures 5J-P).

### 3.14 Hematoxylin-eosin staining

Histological examination of the hippocampal dentate gyrus (DG) region revealed that granule cells in both the control and miR-NC groups displayed intact morphology. In contrast, the miR-618 group exhibited cellular damage, characterized by nuclear fragmentation, hyperchromasia, and pyknosis in numerous granule cells (Figure 5Q).

## 4 Discussion

RDD imposes a severe clinical and financial burden on its sufferers. The lack of dependable biomarkers for RDD emphasizes the urgent need for objective biomarkers to overcome the reliance on subjective clinical diagnosis and enable targeted interventions. Encapsulated in the lipid bilayer membrane of exosomes, miRNAs are protected from degradation by RNA enzymes, and are suitable for clinical detection because of their long-term stable presence in body fluids such as blood, urine, and saliva (Prodan-Bărbulescu et al., 2024a). In the field of disease biomarkers, exosomal miRNAs show great attraction and have become a hot research topic nowadays. Numerous studies have identified biomarkers of depressive disorders (Cuomo-Haymour et al., 2022; Prodan-Bărbulescu et al., 2024a; Chen J. et al., 2025; Gilardi et al., 2025; Liu et al., 2025), however, current studies have not yet reported biomarkers for RDD. Therefore, this study aims to identify candidate miRNA biomarkers for RDD.

In this study, we employed high-throughput sequencing to investigate DEmiRNAs in 3 RDD patients compared to 3 HCs. A total of 49 DEmiRNAs were identified, of which 5 were upregulated, and 44 were downregulated. Among these, 39 were novel predicted miRNAs, and 10 were known miRNAs. Given the greater reliability of known miRNAs as potential biomarkers, the novel predicted miRNAs were excluded from subsequent analyses, while the 10 known DEmiRNAs were retained for further investigation. Among these 10 known DEmiRNAs, three were upregulated (hsa-miR-223-3p, hsa-miR-451a, and hsa-miR-618), and seven were downregulated (hsa-let-7b-5p, hsa-miR-12136, hsa-miR-203a-3p, hsa-miR-3173-3p, hsa-miR-362-3p, hsa-miR-411-5p, and hsa-miR-942-5p).

It has been reported that miR-223-3p has a functional association with the neuronal dysfunction that occurs after adverse childhood experiences (Schmidt et al., 2024). Adverse childhood experiences represent a significant risk factor for major depressive disorder in adulthood and are associated with adult depressive symptoms (O'Shields et al., 2025). Elevated miR-223-3p levels have been found in animal models of traumatic brain injury (Harrison et al., 2017; Sha et al., 2019) and in hippocampal tissue from human patients with epilepsy secondary to tuberous sclerosis complex (Cukovic et al., 2021). Other studies have established connections between miR-223-3p and peripheral nerve injury (Wang et al., 2021), as well as inflammation-associated gene expression alterations in conditions including autism spectrum disorder (Jyonouchi et al., 2017), multiple sclerosis (Bergman et al., 2013), and inflammation induced by environmental toxicants (Fry et al., 2014).

The expression of hsa-miR-618 is dysregulated in patients with schizophrenia and is associated with autophagy dysfunction in the disorder. (Li et al., 2021). Autophagy, a lysosomal degradation process for intracellular components, plays a critical role in the central nervous system by maintaining neuronal homeostasis. Impairment of autophagy disrupts neuronal homeostasis, leading to aberrant neuronal activity that may contribute to various neuropsychiatric disorders (Rubinsztein et al., 2007).

To better explain the biological functions in which the target genes of DEmiRNAs are involved, we performed KEGG pathway analysis on them and annotated and speculated on the pathways in which these target genes might be involved.

The PI3K-Akt signaling pathway plays a pivotal role in the pathological mechanisms of depressive disorder, and its dysfunction is closely linked to key pathological processes in depression. As a key downstream signaling pathway of brain-derived neurotrophic factor (BDNF), the PI3K-Akt pathway is widely distributed in emotion-related brain regions such as the hippocampus, where it regulates multiple processes, including neurogenesis, synaptic remodeling, and neuroinflammation, which are significantly impaired in depressive states (Guo et al., 2024). Studies have shown that in animal models of depression, decreased activity of the PI3K-Akt pathway leads to reduced expression of synaptic proteins and diminished dendritic complexity, thereby contributing to depression-like behaviors. Conversely, pharmacological or molecular activation of this pathway can reverse these alterations, improve synaptic function and autophagic processes, and subsequently alleviate behavioral symptoms (Ahmed et al., 2023; Shi et al., 2024). Thus, the PI3K-Akt pathway not only operates downstream of BDNF signaling but also serves as a critical hub regulating synaptic plasticity and neural adaptation, with its functional status directly influencing the development and reversal of depressive pathology.

The MAPK signaling pathway represents a key intracellular signal transduction cascade involved in cellular stress responses, survival regulation, and neuroinflammatory processes. In depressive disorder, as a major environmental risk factor, chronic stress activates the MAPK pathway, leading to neuroinflammation and thereby promoting the pathology of depressive disorder (Geng et al., 2024). In animal models, chronic stress activates downstream effectors of the MAPK pathway, such as MSK1 and MSK2, which participate in the BDNF-CREB signaling cascade, a central target in antidepressant therapy. Aberrant expression of MSK disrupts neural plasticity and cell survival, aggravating depression-like behaviors (Ji et al., 2022). Furthermore, the MAPK pathway exhibits convergent interactions with other signaling pathways in depressive disorder. Diverse molecular alterations, such as neuroinflammation and synaptic dysfunction, ultimately converge on the MAPK cascade, underscoring its hub-like role in depressive pathology (Li et al., 2023). The MAPK pathway has been identified as an important target for antidepressant intervention. For example, research has demonstrated that the traditional herbal formula Jiao-tai-wan alleviates inflammatory states and improves depressive symptoms in a mouse model of chronic restraint stress by inhibiting MAPK signaling (Tang et al., 2022). Thus, the MAPK pathway acts as a “signal integrator” in depressive disorder, translating environmental stress into neuroinflammation and synaptic impairment. Its dysregulation may constitute a core link in the multifactorial etiology of depressive disorder, highlighting the therapeutic potential of developing specific MAPK inhibitors to block this pathological cascade in future treatments.

The Wnt signaling pathway is an evolutionarily conserved pathway governing development and homeostasis, primarily regulating neurogenesis, synaptic plasticity, and cell survival through the β-catenin-dependent canonical cascade. In depressive disorder, dysregulation of Wnt signaling has been established as a key pathological mechanism, particularly linked to hippocampal dysfunction. Critically, inactivation of Wnt signaling reduces hippocampal neurogenesis and disrupts synaptic transmission, processes that directly contribute to cognitive deficits and affective disturbances characteristic of depressive disorder (Ye et al., 2023; Han et al., 2024; Chen X. et al., 2025). Dysregulation of the Wnt pathway is also associated with miRNA (e.g., miR-128) modulation. miR-128 targets core components of the Wnt cascade, altering downstream gene expression and inducing depression-like behaviors (Verma et al., 2025). Furthermore, Wnt pathway activation exhibits significant antidepressant potential. It has been found that the compound crocin enhances hippocampal neurogenesis by restoring Wnt/β-catenin signaling, thereby ameliorating depressive symptoms in chronic unpredictable mild stress (CUMS) models (Tao et al., 2023). Given the tunable nature of Wnt signaling, targeted interventions represent a promising novel antidepressant strategy, particularly for treatment-resistant depression with high translational value.

Using RT-qPCR, we validated that the expression levels of miR-618 and miR-223-3p in plasma exosomes from RDD patients were significantly higher than those in HCs, which was consistent with the results obtained from high-throughput sequencing. Based on the relative expression levels derived from RT-qPCR, ROC curve analysis was performed to evaluate the diagnostic performance of miR-618 and miR-223-3p.

The ROC curve is a visual tool used to assess the performance of diagnostic models. Its fundamental principle involves ranking predicted values of cases and non-cases from lowest to highest, thereby establishing a series of cutoff points. The AUC represents the area enclosed by the ROC curve. A higher AUC indicates better overall discriminative ability of the model. Specifically, an AUC >0.9 suggests very high accuracy, enabling the model to effectively distinguish cases from non-cases; an AUC between 0.7 and 0.9 indicates good accuracy with potential clinical utility; and an AUC between 0.5 and 0.7 suggests limited accuracy and minimal clinical value. Sensitivity, also known as the true positive rate, reflects the model’s ability to correctly identify positive cases. Higher sensitivity implies a lower probability of missing a disease. Specificity, or the true negative rate, reflects the model’s ability to correctly identify negative cases. Higher specificity indicates a lower probability of misdiagnosis. The AUC values for miR-618 and miR-223–3p were 0.782 and 0.762, respectively, indicating good potential diagnostic performance. Therefore, these miRNAs can be considered candidate biomarkers for RDD. Targeting both miRNAs collectively may offer more effective therapeutic interventions or diagnostic strategies.

MiRNA mimics are synthetic short double-stranded RNA molecules that mimic the structure and function of endogenous mature miRNAs, enabling overexpression of specific miRNAs in cells or tissues (Zhang et al., 2023). Lacking cell-membrane permeability, miRNA mimics require transfection reagents to form complexes for cellular internalization. Moreover, they cannot cross the blood-brain barrier (BBB) independently, which limits their *in vivo* application (Wang et al., 2023). Exosomes, which possess a stable lipid bilayer and inherent BBB-penetrating ability, are widely used as drug delivery vehicles (Song et al., 2020). Among various cell sources, HEK293 T cells exhibit rapid growth, active secretory pathways, and high exosome productivity. They also show exceptionally high transfection efficiency, allowing easy introduction of plasmids, miRNAs, siRNAs, and other regulatory elements, followed by efficient packaging of the desired molecules into secreted exosomes, thereby enabling precise engineering of exosome content and function (Huang et al., 2024). Based on this rationale, we isolated exosomes from HEK293 T cells transfected with miR-618 mimics, which were thus loaded with miR-618 mimics. Successful construction of the miR-618 delivery vehicle was confirmed by RT-qPCR detection of elevated miR-618 levels in exosomes and by uptake assays in BV2 cells.

Previous bioinformatics analysis identified TP53 and CCND1 as both target genes of miR-618 and hub genes in the PPI network, with the PI3K-Akt signaling pathway being one of their downstream cascades. To validate the function of miR-618, we achieved its overexpression in brain tissue via targeted delivery of miR-618 mimics and then examined its impact on the PI3K-Akt pathway. RT-qPCR confirmed increased expression of miR-618 in the mouse hippocampus, along with downregulation of its target genes TP53 and CCND1. Behavioral tests demonstrated that miR-618 could induce depression-like behaviors in mice. Decreased levels of p-PI3K/t-PI3K and p-Akt/t-Akt indicated that miR-618 regulates the PI3K-Akt signaling pathway. Reduced BDNF expression suggested impaired synaptic plasticity. Increased Bax, decreased Bcl2, and histopathological alterations in the DG region collectively pointed to neuronal damage. Therefore, we propose that miR-618 contributes to depression-like behaviors by regulating the PI3K-Akt signaling pathway and subsequently inducing neuronal and synaptic plasticity impairment.

It is worth acknowledging that there are some limitations of this study. The high-throughput sequencing included only 3 RDD patients and 3 HCs, reducing statistical power and increasing the risk of false-positive or false-negative results. The scarcity of RDD patient samples limited the sample size used for RT-qPCR validation (18 RDD patients and 18 HCs), which in turn constrained the reliability of the results. Due to the lower reliability of the newly discovered miRNAs compared to the already identified miRNAs, they were not included in the subsequent studies.

This study is a preliminary discovery and validation of biomarkers, aiming to provide high-quality candidate biomarkers for subsequent translational research. The design of this study and the calculation of statistical power were based on the overall sample of subjects, so no gender-based analysis was conducted. This study lacks a prospective suspected cohort and did not conduct blind testing. Therefore, the current research design cannot support the conclusion that these miRNA markers have direct diagnostic utility. The longitudinal tracking of miRNA expression during the relapse or remission phases of RDD was not conducted, which hindered the in-depth understanding of the dynamic changes of biomarkers. To address these limitations, future research efforts should focus on deepening and expanding upon the current study. Firstly, in large-scale prospective multicenter cohorts, blind detection should be employed to further validate the diagnostic performance and stability of these candidate biomarkers, and gender-based stratified analyses should be carried out to assess their universality and potential gender-specific differences. Secondly, a long-term clinical follow-up cohort should be established to collect longitudinal samples from patients, and the changes in miRNA expression profiles at different disease stages should be dynamically monitored, which is crucial for evaluating prognostic value and as a potential therapeutic monitoring marker.

This study has certain limitations in elucidating the mechanistic role of miR-618. While we have preliminarily established the involvement of the PI3K-Akt pathway, whether miR-618 influences other related pathways and the potential crosstalk among them remains to be validated in future experiments. Furthermore, our investigation relied primarily on *in vivo* models; functional validation at the cellular level was not included. Future studies integrating cellular models would be valuable to provide deeper mechanistic insights.

## 5 Conclusion

This study conducted the first investigation into the miRNA expression profile within plasma exosomes of RDD patients, identifying 49 DEmiRNAs. Through GO and KEGG pathway enrichment analyses, we conducted preliminary investigations into the functional characteristics and mechanistic pathways associated with the DEmiRNAs. Subsequent RT-qPCR and ROC curve analysis identified miR-618 and miR-223–3p in plasma exosomes as candidate non-invasive biomarkers for detecting RDD. Functional validation *in vivo* further indicated that miR-618 contributes to depression-like behaviors by regulating the PI3K-Akt pathway and subsequently inducing neuronal and synaptic plasticity impairment.

## Data Availability

The raw sequence data reported in this paper have been deposited in the Genome Sequence Archive (Genomics, Proteomics & Bioinformatics 2021) in National Genomics Data Center (Nucleic Acids Res 2024), China National Center for Bioinformation/Beijing Institute of Genomics, Chinese Academy of Sciences (GSA-Human: HRA011479), which are publicly accessible at https://ngdc.cncb.ac.cn/search/specific?db=hra&q=HRA011479.
