# Supplementary material for: Identification and functional characterization of plasma exosomal miR-618 as a novel biomarker in recurrent depressive disorder
Source: Front Pharmacol. 2026 Jun 1;17:1816232. doi: 10.3389/fphar.2026.1816232 (PMC13265561; doi:10.3389/fphar.2026.1816232)
Supplement: Supplementary file 1 [file Supplementaryfile1.docx]

Supplementary Material

**1 Materials and methods**

**1.1 MiRNA sequencing and different expression analysis**

The RNA concentration of each sample was measured using a NanoDrop ND-1000 instrument (Thermo Fisher, USA). The RNA Library was constructed using the GenSeq Small RNA Library Prep Kit (GenSeq, China) according to vendor instructions. After cutting gel to recover pieces of a specific size, followed by quality control and quantification of the library using the BioAnalyzer 2100 system (Thermo Fisher, USA). Sequencing was carried out on Illumina NovaSeq (Illumina, USA) with 50bp single-end mode. Raw data were generated after sequencing, image analysis, base calling and quality filtering on sequencer. Firstly, Q30 was used to perform quality control. The adaptor sequences were trimmed and the adaptor-trimmed-reads (≥15nt) were left by cutadapt software (v1.9.3). The trimmed reads were aligned to the merged pre-miRNA databases (known pre-miRNA from miRBase plus the newly predicted pre-miRNAs) using Novoalign software (v3.02.12) with at most one mismatch. The numbers of mature miRNA mapped tags were defined as the raw expression levels of that miRNA. The read counts were normalized by TPM (tag counts per million aligned miRNAs) approach. Then, trimmed reads from all samples were pooled, and miRDeep2 software (v2.0.0.5) was used to predict novel miRNAs. DEmiRNAs between two groups were filtered by Fold Change and P-value.

**1.2 RT-qPCR**

According to the operating instructions of the BIOG miRNA Stem-loop RT Kit (BAIDAI, China) and BIOG miRNA Stem-loop SYBR qPCR Kit (BAIDAI, China), RNase-free 1.5 mL centrifuge tubes were used to prepare the RT reaction mixture by combining the following components: Total RNA (100 ng), BIOG RTase Mix (2 μL), 2× RT Buffer (10 μL), 1 μL of RT Primer (2 μM), and RNase-free ddH₂O to a final volume of 20 μL. After centrifuging the tubes at 5000 rpm for 5 seconds, the reaction was carried out sequentially at 25°C for 5 min, 50°C for 15 min, and 85°C for 5 min.

Following the completion of the reverse transcription reaction, the tubes were centrifuged again at 5000 rpm for 5 seconds, then quickly placed on ice. Subsequently, the following mixture was prepared in a 96-well PCR plate (Service, China): Template DNA (2 μL), 2× BIOG miRNA SYBR Mastermix (10 μL), 0.4 μL of Forward primer (10 μM), 0.4 μL of Reverse primer (10 μM), and ddH₂O to a final volume of 20 μL. The PCR plate was sealed with sealing film and loaded into the LightCycler 96 Real-Time PCR instrument (Roche, Switzerland) for PCR amplification and fluorescence signal collection.

The relative gene expression levels were calculated using the 2^-ΔΔCq^ method: ΔCq=Cq(target gene)-Cq(U6), ΔΔCq=ΔCq(RDD group)-ΔCq (HC group), Relative expression=2^-ΔΔCq^. Here, Cq represents the cycle number at which the real-time fluorescence intensity reaches the predefined threshold during amplification.

**1.3 Cell transfection**

Upon reaching approximately 70% confluence, the medium of 293T cells in the T75 flask was replaced with 8500 μL of Opti-MEM medium (Byotime, China). After 1 h of incubation, transfection complexes were prepared under light-protected conditions. Briefly, 750 μL of Opti-MEM was added into each of two 1.5 mL tubes (designated A and B). Tube A received 30 μL of Lipo293 Plus Transfection Reagent (Byotime, China), while Tube B received 30 μL of 20 μM cy3-labeled miR-NC mimics or cy5-labeled miR-618 mimics (synthesized by Fuzhou Zaiji Biotechnology Co., Ltd., China). Each mixture was gently pipetted to mix and then incubated for 5 min. Subsequently, the contents of Tubes A and B were combined, mixed thoroughly, and incubated for an additional 15 min to allow complex formation. The final complex solution was carefully added dropwise to the target 293T cells. Control cells did not undergo transfection. All cells were then returned to the incubator. 6 h post-transfection, the medium was replaced with 10 mL of fresh DMEM medium containing 10% exosome-depleted fetal bovine serum (Umibio, China). After a further 24-h incubation, the cell culture supernatant was collected for exosome extraction.

**1.4 Verification of cell transfection efficiency**

An appropriate number of 293T cells were seeded into confocal dishes (NEST, China) and cultured overnight. When the cells reached approximately 70% confluence the next day, the culture medium was replaced with 1750 μL of Opti-MEM medium. After 1 h of incubation, transfection complexes were prepared under light-protected conditions. Briefly, 750 μL of Opti-MEM was aliquoted into each of two sterile 1.5 mL microcentrifuge tubes (labeled A and B). Tube A was supplemented with 5 μL of Lipo293 Plus Transfection Reagent, while Tube B received 5 μL of 20 μM cy3-labeled miR‑NC mimics or cy5-labeled miR‑618 mimics. After gentle pipetting, each mixture was incubated for 5 min. The solutions from Tubes A and B were then combined, mixed thoroughly, and incubated for an additional 15 min to allow complex formation. The resulting mixture was carefully added dropwise to the 293T cells intended for transfection. Cells in the control group were treated identically but without transfection reagents. All dishes were returned to the incubator for 6 h.

After incubation, the old medium was aspirated and cells were washed three times with phosphate buffer saline (PBS). Subsequently, cells were fixed with 1 mL of 4% paraformaldehyde (Biosharp, China) for 20 min at room temperature. Following fixation, cells were washed three times with PBS and stained with 200 μL of DAPI solution (10 μg/mL, Meilunbio, China) for 5 min. After removal of the DAPI solution, cells were washed three times with PBS. Imaging was performed using a laser scanning confocal microscope (Leica, Germany) in dual‑channel mode. DAPI was excited at 405 nm; cy3 was excited at 550 nm; and cy5 was excited at 650 nm. Corresponding fluorescence images were captured for analysis.

**1.5 Western blot for hippocampal tissue samples**

The hippocampus were homogenized in pre-chilled RIPA lysis buffer containing 1mM PMSF (LABLEAD, China). The homogenates were then centrifuged at 12,000 ×g for 20 min at 4°C. The resulting supernatant, containing the total protein fraction, was collected for subsequent analysis. The protein concentration of each sample was determined using the BCA Protein Concentration Assay Kit (LABLEAD, China), following the manufacturer's instructions. Subsequently, protein samples were mixed with one-fourth volume of 5× SDS-PAGE protein loading buffer (Servicebio, China) and denatured by heating at 95°C for 5 min.

20 μg of protein was separated on a 10% SDS-PAGE gel (pre-cast using a One Step PAGE Gel Super Fast Preparation Kit, Meilun, China) by SDS-PAGE under constant voltage (200 V) for 45 min, followed by being electrophoretically transferred to a polyvinylidene fluoride (PVDF) membrane (Immobilon, Germany) at 400 mA for 25 min. After incubation for 1 h at room temperature with Tris-Buffered Saline with Tween 20 (TBST, Servicebio, China) containing 5% Bovine Serum Albumin (Beyotime, China), the PVDF membrane was rinsed three times with TBST for 10 min each, followed by overnight incubation at 4℃ with the following primary antibody: BDNF Recombinant Rabbit Monoclonal Antibody (Clone NO. SJ12-09, Cat# ET1606-42, 1:5000, HUABIO, China), Bax Recombinant Rabbit Monoclonal Antibody (Clone NO. R03-1D3, Cat# R22708, 1:5000, zenbio, China), Bcl2 Recombinant Rabbit Monoclonal Antibody (Clone NO. R07-2A1, Cat# R23309, 1:500, zenbio, China), Phospho-PI3K Recombinant Rabbit Monoclonal Antibody (Clone NO. PSH01-38, Cat# HA721672, 1:1000, HUABIO, China), PI3 Kinase p110 beta Recombinant Rabbit Monoclonal Antibody (Clone NO. JE65-38, Cat# HA722474, 1:1000, HUABIO, China), Phospho-AKT Recombinant Rabbit Monoclonal Antibody (Clone NO. 2E17, Cat# 80455-1-RR, 1:500, proteintech, China), AKT1/2/3 Recombinant Rabbit Monoclonal Antibody (Clone NO. ST48-09, Cat# ET1609-51, 1:5000, HUABIO, China), Beta Actin Recombinant Rabbit Monoclonal Antibody (Clone NO. 4H1, Cat# 81115-1-RR, 1:5000, HUABIO, China) and GAPDH Rabbit Monoclonal Antibody (Clone NO. R09-4E-1, Cat# R380626, 1:10000, zenbio, China). The following day, the PVDF membrane was rinsed three times with TBST for 10 min each, and then incubated with HRP-Labelled Goat Anti-Rabbit IgG (H+L) (Cat# A0208, 1:1000, Beyotime, China) for 1 h at room temperature. After incubation, the PVDF membrane was rinsed again three times with TBST for 10 min each. Finally, the PVDF membrane was infiltrated with BeyoECL Plus (Beyotime, China), followed by a protein blotting assay using the e-BLOT Touch Imager System (e-BLOT, China).

**List of abbreviations**

| **Abbreviation** | **Full name** |
| --- | --- |
| PBS | phosphate buffer saline |
| SDS-PAGE | sodium dodecyl sulfate polyacrylamide gel electrophoresis |
| PVDF | polyvinylidene fluoride |
| TBST | Tris-Buffered Saline with Tween 20 |

**Table S1 Differentially expressed serum exosomal miRNAs between the RDD and HC groups**

| **Mature-miRNA** | **Mature-sequence** | **Fold Change** | **P-value** | **FDR** | **Regulation** |
| --- | --- | --- | --- | --- | --- |
| hsa-miR-223-3p | UGUCAGUUUGUCAAAUACCCCA | 6.810878943 | 0.002672254 | 0.416775058 | up |
| hsa-miR-451a | AAACCGUUACCAUUACUGAGUU | 3.201916959 | 0.014672086 | 0.416775058 | up |
| hsa-miR-618 | AAACUCUACUUGUCCUUCUGAGU | 151.0721075 | 0.01222614 | 0.416775058 | up |
| hsa-miR-novel-chr8_39308 | GAUGUGGAAUGUUUAAUCUAU | 75.37961105 | 0.03801941 | 0.416775058 | up |
| hsa-miR-novel-chr8_40463 | CAUCUGUGGGAUUAUGACUGAAC | 34.62240549 | 0.018172547 | 0.416775058 | up |
| hsa-let-7b-5p | UGAGGUAGUAGGUUGUGUGGUU | 0.287341073 | 0.026041366 | 0.416775058 | down |
| hsa-miR-12136 | GAAAAAGUCAUGGAGGCC | 0.003041635 | 0.019197061 | 0.416775058 | down |
| hsa-miR-203a-3p | GUGAAAUGUUUAGGACCACUAG | 0.00356887 | 0.023912886 | 0.416775058 | down |
| hsa-miR-3173-3p | AAAGGAGGAAAUAGGCAGGCCA | 0.00390669 | 0.026718892 | 0.416775058 | down |
| hsa-miR-362-3p | AACACACCUAUUCAAGGAUUCA | 0.003819721 | 0.02606034 | 0.416775058 | down |
| hsa-miR-411-5p | UAGUAGACCGUAUAGCGUACG | 0.010970097 | 0.040925213 | 0.416775058 | down |
| hsa-miR-942-5p | UCUUCUCUGUUUUGGCCAUGUG | 0.009085831 | 0.03269092 | 0.416775058 | down |
| hsa-miR-novel-chr10_1635 | GCGGCCCGGGGUGGAGAGA | 0.004778634 | 0.029975032 | 0.416775058 | down |
| hsa-miR-novel-chr11_2678 | UAUCCGCUGGCUUACUCUCUCU | 0.00306821 | 0.019441246 | 0.416775058 | down |
| hsa-miR-novel-chr12_4629 | GGUGGGCGCGGCGCGGGC | 0.003997707 | 0.027406555 | 0.416775058 | down |
| hsa-miR-novel-chr12_5365 | GGAGGAGGAGGAGGAAG | 0.00556205 | 0.03107392 | 0.416775058 | down |
| hsa-miR-novel-chr14_8504 | CUGGGCUGGGCUCCUCUG | 0.003369582 | 0.022201721 | 0.416775058 | down |
| hsa-miR-novel-chr15_8879 | CGGGGCAGGGCAGGGAAGGGAA | 0.004298492 | 0.028971547 | 0.416775058 | down |
| hsa-miR-novel-chr15_8981 | AGUGGGAAAGGAUGUGG | 0.002771379 | 0.016683244 | 0.416775058 | down |
| hsa-miR-novel-chr16_11304 | AGGCUGAGAACAUGAGUAU | 0.002863309 | 0.017541343 | 0.416775058 | down |
| hsa-miR-novel-chr17_13999 | ACUGGCAUUGUCAUGGACUCUGG | 0.00306821 | 0.019441246 | 0.416775058 | down |
| hsa-miR-novel-chr1_17509 | AGAAGGGAGGGGCUGGGAAGAAGGG | 0.003242204 | 0.021034524 | 0.416775058 | down |
| hsa-miR-novel-chr1_20267 | GUGGGGCCUGGGAGGGG | 0.003014286 | 0.018949747 | 0.416775058 | down |
| hsa-miR-novel-chr20_21362 | UGCUGAAAGCCGUUUCCCGUGU | 0.003182056 | 0.020482572 | 0.416775058 | down |
| hsa-miR-novel-chr20_21854 | UGGGAGGCUGUGAAACUG | 0.002816287 | 0.017105288 | 0.416775058 | down |
| hsa-miR-novel-chr22_24122 | UGGGCUGGGAGGCAGAGCG | 0.00306821 | 0.019441246 | 0.416775058 | down |
| hsa-miR-novel-chr22_24316 | GGGGACGGGAGUGGGACUGG | 0.00248922 | 0.014170665 | 0.416775058 | down |
| hsa-miR-novel-chr2_25083 | GAUGUUAGCUGAAUCCUCUCAU | 0.007187621 | 0.03208765 | 0.416775058 | down |
| hsa-miR-novel-chr2_25720 | UCACUCUGUAGACCAGGCU | 0.002411535 | 0.01349243 | 0.416775058 | down |
| hsa-miR-novel-chr2_26004 | GAGGAGGAGGAAGAAGAAGG | 0.002770783 | 0.016684715 | 0.416775058 | down |
| hsa-miR-novel-chr2_26912 | GAUGUUAGCUGAAUCCUCUCAU | 0.007187621 | 0.03208765 | 0.416775058 | down |
| hsa-miR-novel-chr2_27269 | UGGGAUUUGAACUCAAGA | 0.003736536 | 0.025396117 | 0.416775058 | down |
| hsa-miR-novel-chr2_27602 | GAGAGUUACUGGUUCGAAUC | 0.003124097 | 0.019951845 | 0.416775058 | down |
| hsa-miR-novel-chr3_27824 | CAGCUGGAGGUGCUGUGGG | 0.002684045 | 0.015887142 | 0.416775058 | down |
| hsa-miR-novel-chr3_29001 | UGAGGAUGCAGUUCUCCUUUGAG | 0.003656894 | 0.024702339 | 0.416775058 | down |
| hsa-miR-novel-chr3_29002 | UGAGGAUGCAGUUCUCCUUUGAG | 0.003656894 | 0.024702339 | 0.416775058 | down |
| hsa-miR-novel-chr4_30389 | GGGAGCCUGAGAAAUGGC | 0.003182056 | 0.020482572 | 0.416775058 | down |
| hsa-miR-novel-chr5_32335 | UUGGCUGGAGACCUGGUG | 0.003242204 | 0.021034524 | 0.416775058 | down |
| hsa-miR-novel-chr5_32550 | AAAUGACUGGUGGGUGAGCA | 0.003369582 | 0.022201721 | 0.416775058 | down |
| hsa-miR-novel-chr5_32893 | CAUGAGUUGGAGUGUAGG | 0.002911927 | 0.017993657 | 0.416775058 | down |
| hsa-miR-novel-chr5_34040 | GAGAAUCACUUGAACCC | 0.001579646 | 0.005805458 | 0.416775058 | down |
| hsa-miR-novel-chr6_35532 | GGCGGGUCGGGGCGGGGG | 0.005554203 | 0.031001177 | 0.416775058 | down |
| hsa-miR-novel-chr6_36159 | CAGGGAGAGGAGGUAGAGGG | 0.006153482 | 0.031523569 | 0.416775058 | down |
| hsa-miR-novel-chr6_36163 | CAGGGAGAGGAGGUAGAGGG | 0.006153482 | 0.031523569 | 0.416775058 | down |
| hsa-miR-novel-chr6_36210 | AGAAUUGCGUUUGGACAAUCA | 0.009606367 | 0.034725087 | 0.416775058 | down |
| hsa-miR-novel-chr6_36858 | AGAAGCGCAUCACUGAGGCAGAGAA | 0.003736536 | 0.025396117 | 0.416775058 | down |
| hsa-miR-novel-chr7_37266 | UGGGAUUUGAACUUCAG | 0.003997707 | 0.027406555 | 0.416775058 | down |
| hsa-miR-novel-chr7_38115 | AACAAGAGUGAAGUUCUGUCU | 0.003124097 | 0.019951845 | 0.416775058 | down |
| hsa-miR-novel-chrX_43483 | UCCUUGAUCCUCUGCCA | 0.006635351 | 0.009975493 | 0.416775058 | down |

**Mature miRNA**: the name of mature miRNA (miRNAs with ‘novel’ are newly discovered miRNAs); **Mature-sequence**: the sequence of mature miRNA; **Fold Change**: the fold change of miRNA expression in RDD relative to HC; **P-value**: nominal P-value between two groups of samples; **FDR**: correction for P-value using the false discovery rate (FDR) according to the Benjamini-Hochberg method; **Regulation**: “up” means up-regulation, “down” means down-regulation.
